# Supplementary material for: Climate-induced phenology shifts linked to range expansions in species with multiple reproductive cycles per year
Source: Nat Commun. 2019 Oct 24;10:4455. doi: 10.1038/s41467-019-12479-w (PMC6813360; doi:10.1038/s41467-019-12479-w)
Supplement: Supplementary file 1 — Supplementary Information [file 41467_2019_12479_MOESM1_ESM.pdf]

Supplementary Information

Climate-induced phenology shifts linked to range expansions in species with multiple reproductive cycles per year

Macgregor *et al.*

Supplementary Figures 1–7

Supplementary Tables 1–6

Supplementary Figures

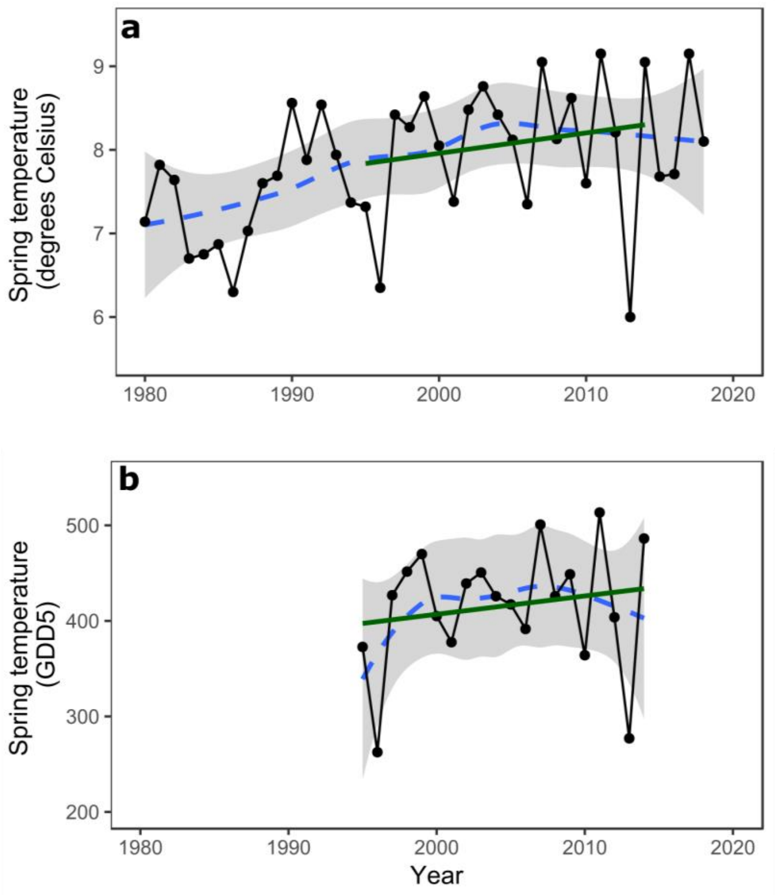

Supplementary Figure 1 | Trends in UK spring temperature before, during and after our study period (1995-2014). Points show **a**, national mean spring daily temperature (°C), and **b**, mean accumulated growing degree days above a 5 °C threshold (GDD5) across the 141 sites included in the study. Blue dashed lines with grey 95% confidence interval show smoothed trends fitted by a generalized additive model. Green lines show trends over the study period 1995-2014 fitted by a linear regression. Data for **a** from the UK Meteorological Office (https://www.metoffice.gov.uk/climate/uk/summaries/datasets), and data for **b** extracted as described in the methods.

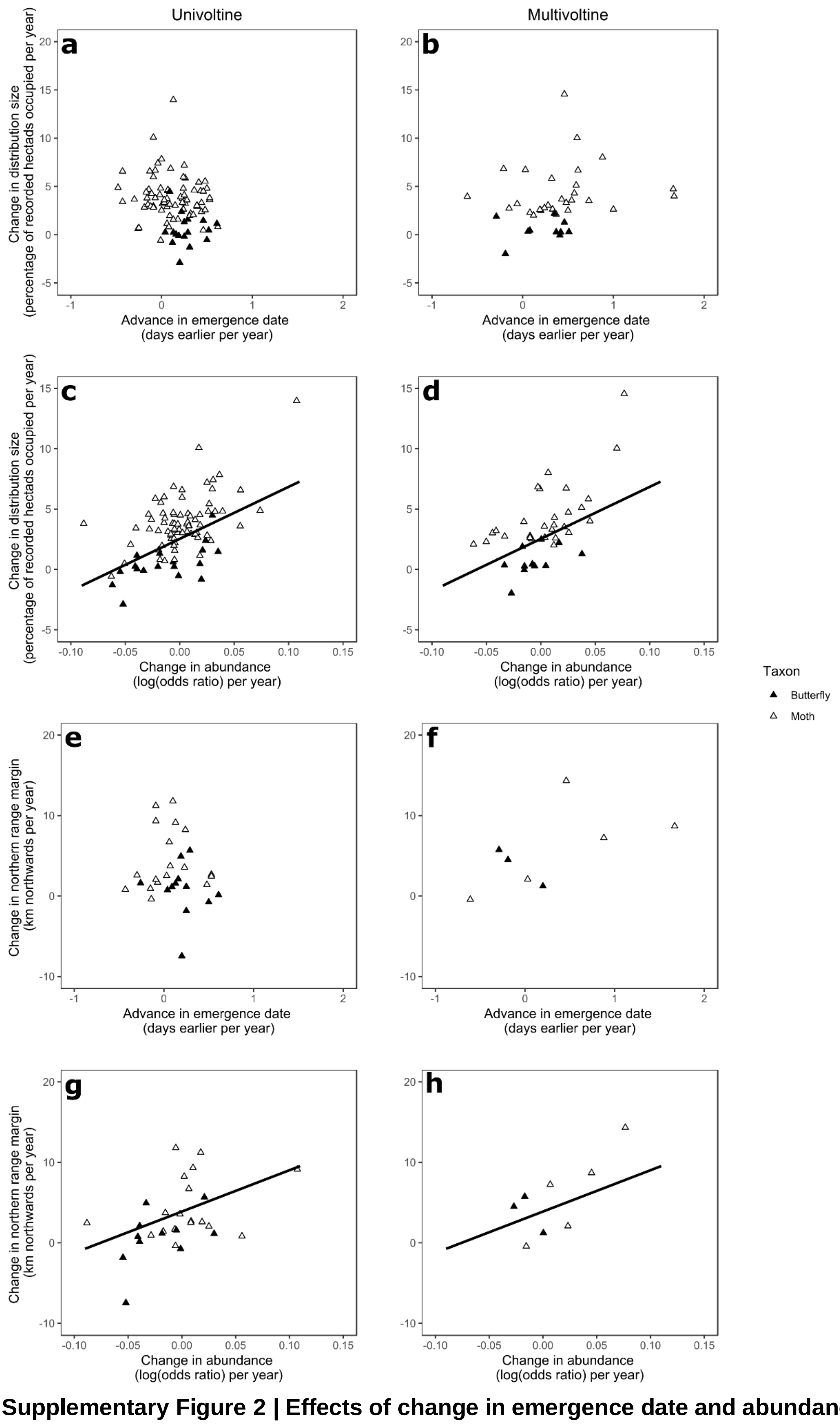

Supplementary Figure 2 | Effects of change in emergence date and abundance on trends in distribution size and latitude of northern range margins do not depend on

species' voltinism (Table 1). Effects are shown of advance in emergence date on **a**,**b**, distribution trends and **e**,**f**, northern range margin, and effects of abundance change on **c**,**d**, distribution trends and **g**,**h**, northern range margin. Points show trends over the study period (1995-2014) at species-level for univoltine (**a**,**c**,**e**,**g**) and multivoltine (**b**,**d**,**f**,**h**) species, and are filled according to taxonomic group (butterflies: filled, moths: open). Lines depict model-predicted relationships between variables, and are plotted only when relationships were significantly different to zero ($P < 0.05$). Advance in phenology is not related to change in distribution size (**a**,**b**) or to change in northern range margin (**e**,**f**). However, change in abundance is positively related to change in distribution size (**c**,**d**) and change in northern range margin (**g**,**h**) for both univoltine and multivoltine species.

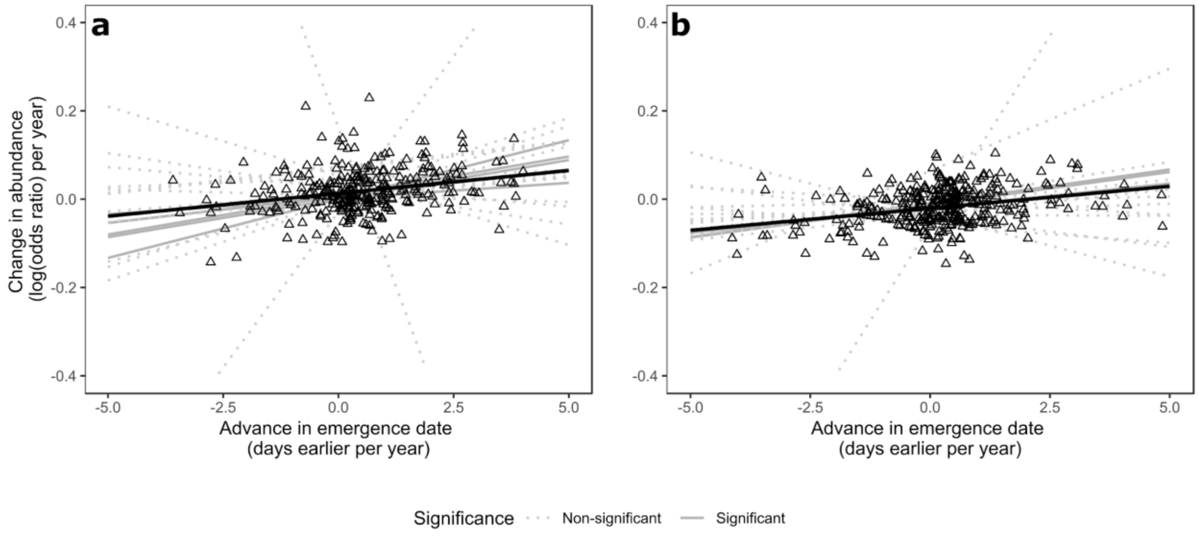

Supplementary Figure 3 | Advancing phenology correlates with increasing abundance at population-level for multivoltine species regardless of species-level abundance trend. Lines depict model-predicted relationships between phenology and abundance trends, from generalised linear mixed-effects models, for multivoltine species with, respectively, (**a**) increasing and (**b**) declining abundance trends at species-level. Overall trends are plotted (solid black lines) where significantly different to zero (*P* < 0.05). Points show advances in phenology and changes in abundance over the study period at population-level. Grey lines show relationships calculated independently for each species in the study; lines are solid if the relationship is significantly different to zero (*P* < 0.05), or otherwise are dotted.

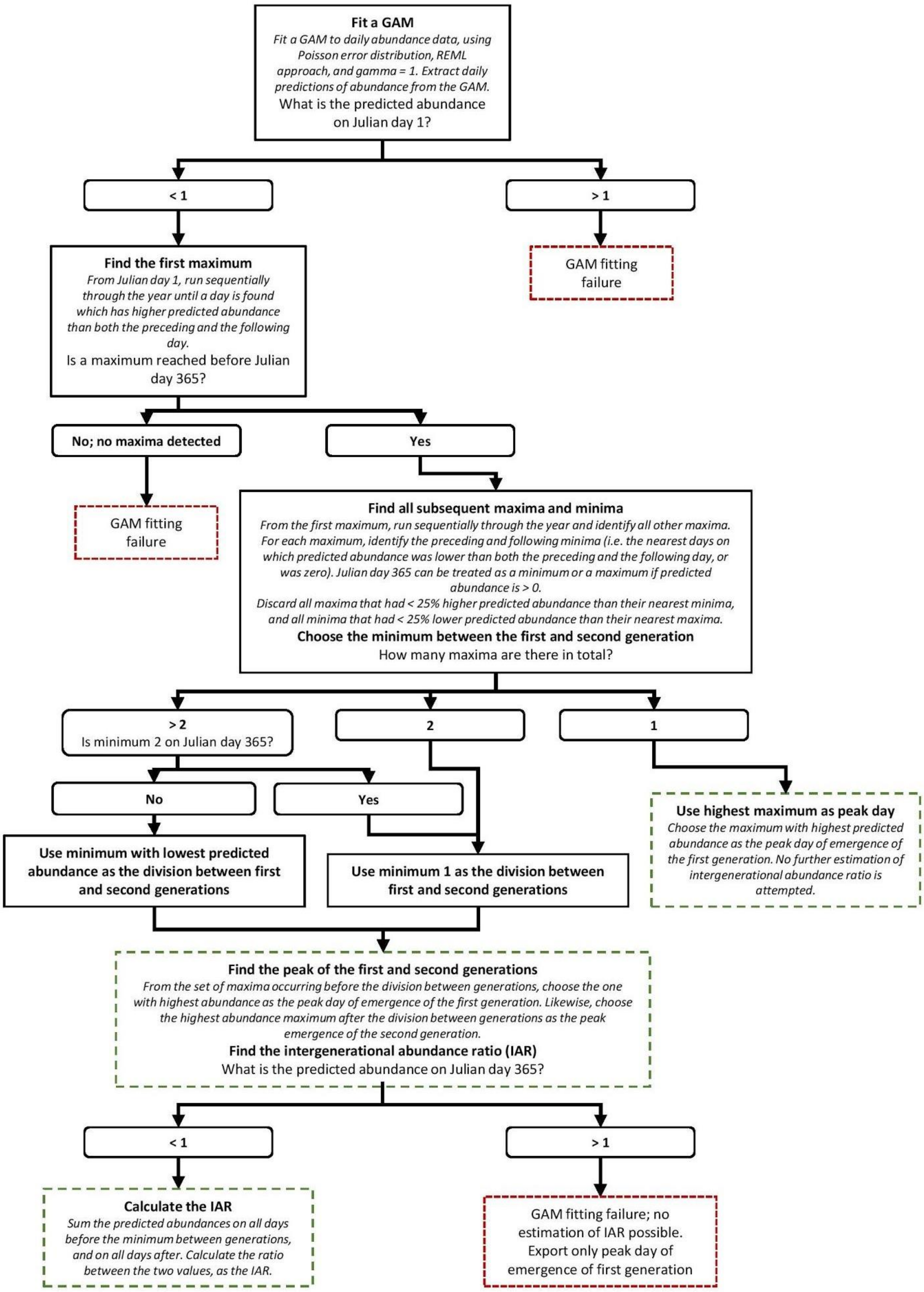

Supplementary Figure 4 | Logical steps to analyse GAMs and extract (i) peak day of emergence of the first generation and (ii) intergenerational abundance ratio (IAR). Boxes outlined in green dashed borders indicate steps where these variables were estimated. Reaching a box outlined in red dotted borders indicates that the fitted GAM did not allow for reliable estimation of one or both variables.

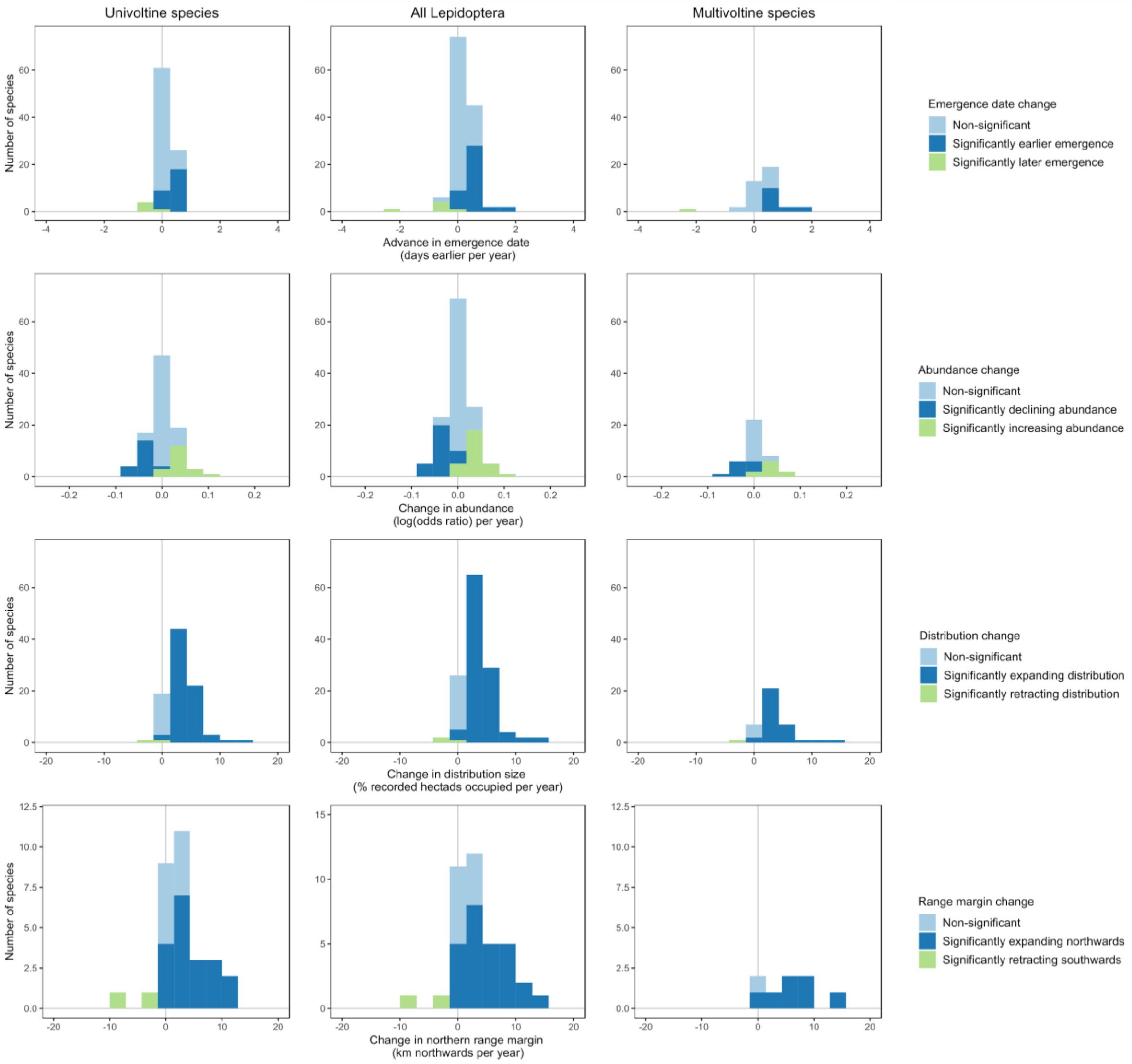

Supplementary Figure 5 | Histograms of species-level rates of change over time, showing differences between univoltine (left) and multivoltine (right) species, and with both combined (centre). Rates of change are show for four variables: advance in first-generation emergence date, change in abundance, change in distribution size, and advance in latitude of northern range margin.

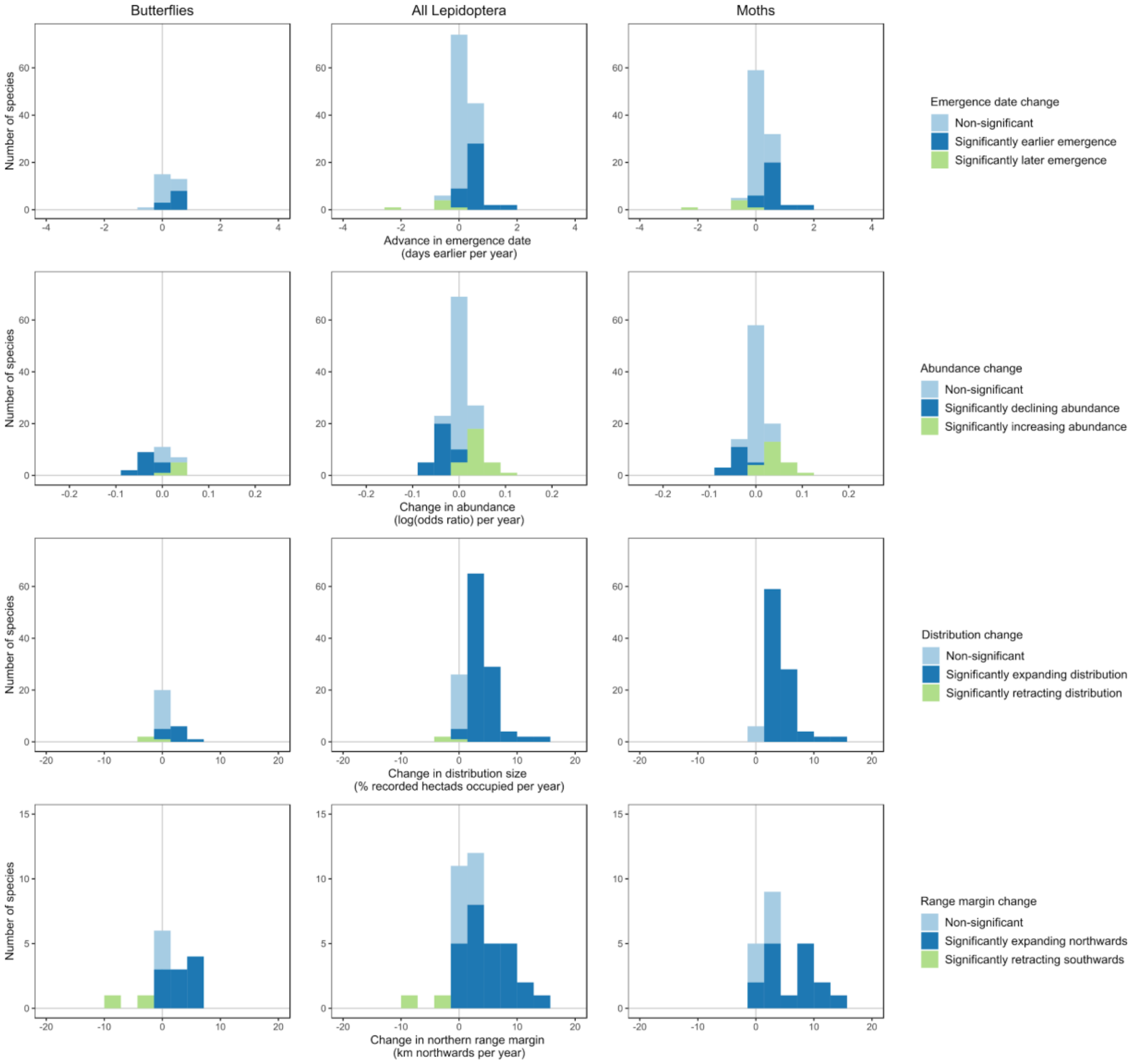

Supplementary Figure 6 | Histograms of species-level rates of change over time, showing differences between butterflies (left) and moths (right), and with both combined (centre). Rates of change are show for four variables: advance in first-generation emergence date, change in abundance, change in distribution size, and advance in northern range margin latitude.

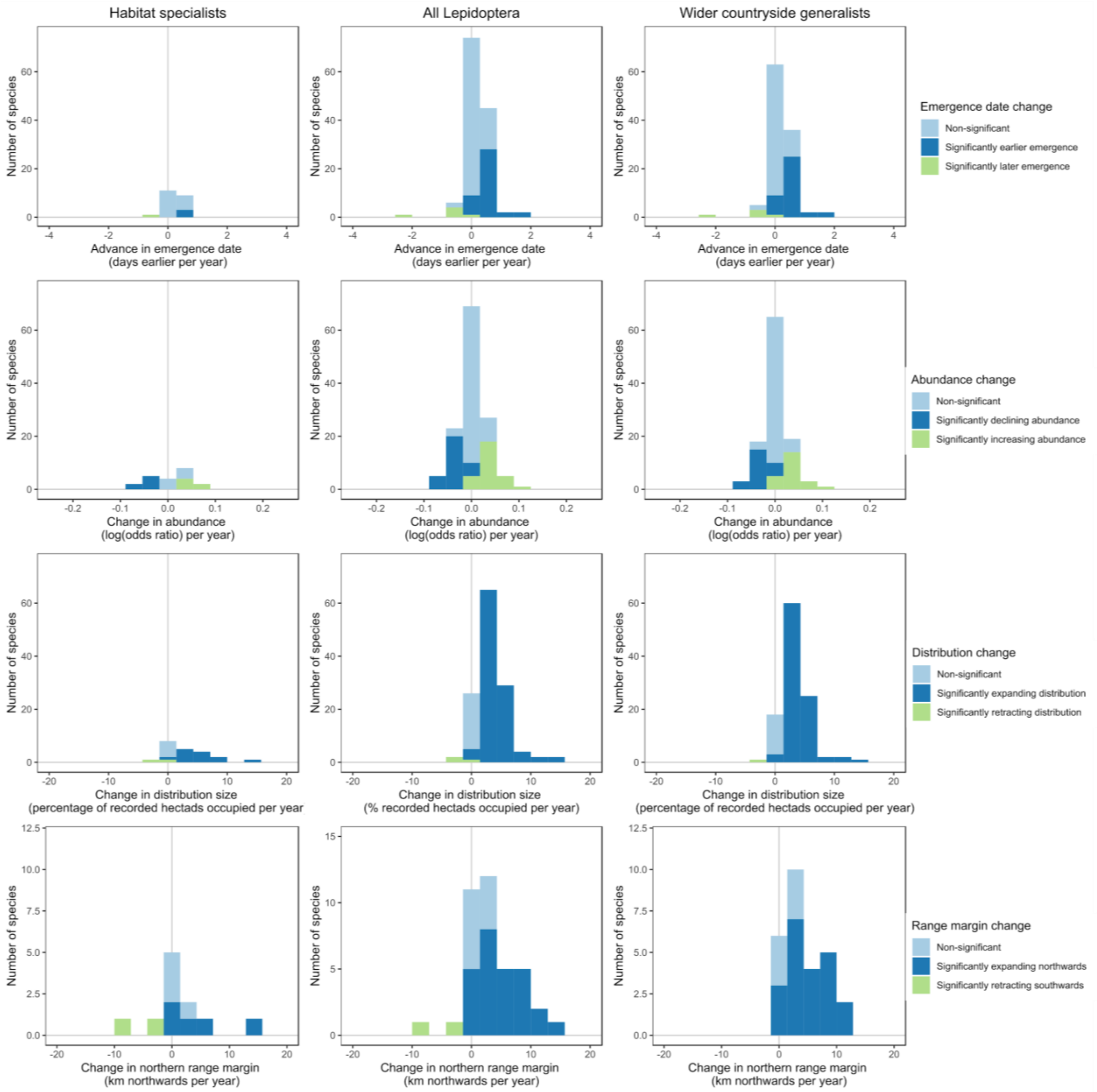

Supplementary Figure 7 | Histograms of species-level rates of change over time, showing differences between habitat specialists (left) and wider countryside generalists (right), and with both combined (centre). Rates of change are show for four variables: advance in first-generation emergence date, change in abundance, change in distribution size, and advance in northern range margin latitude.

Supplementary Tables

Supplementary Table 1 | Summary of data from each recording scheme that passed the data selection procedure.

		Butterflies	Moths
Final species included		29	101
Population monitoring schemes		UKBMS	RIS
	Sites	110	31
	Populations	750	927
	Records	128624	294289
	Individuals	2550081	922594
National distribution recording schemes		BNM	NMRS
	Heavily-recorded hectads	1639	475
	Hectad-level presence records	392918	520119

Supplementary Table 2 | Mean annual change in the four variables (emergence date, abundance, occupied distribution and NRM) over the study period (1995-2014). Results of non-parametric tests are shown testing whether (i) the median rate of change is different to zero; (ii) the median rate of change differs between butterflies and moths; (iii) the median rate of change differs between univoltine and multivoltine species; and (iv) the median rate of change differs between habitat specialist species and wider-countryside generalist species. Non-parametric tests were used because some samples were not normally distributed (according to the results of Shapiro-Wilk normality tests). For one-sample tests, Wilcoxon signed-rank tests were applied with the null hypothesis that μ = 0, and the test statistic (TS) was v. For two-sample tests, Wilcoxon rank sum tests were applied with the null hypothesis that the two samples were equal, and the TS was w. Tests that had statistical significance ($P < 0.05$) are indicated in bold.

Variable	Mean annual change over all species (s.e.)	H1: mean change is different to zero		H1: change is different between butterflies and moths		H1: change is different between univoltine and multivoltine species		H1: change is different between habitat specialists and wider countryside generalists	
		TS	*P*	TS	*P*	TS	*P*	TS	*P*
Advance in emergence date (days per year)	0.19 (0.04)	6772	**<0.001**	1650.5	0.300	1209.5	**0.004**	1292	0.352
Increase in abundance (change in log odds ratio per year)	0.0003 (0.003)	4294.5	0.932	1099	**0.041**	1676.5	0.620	1216	0.653
Increase in occupied distribution (change in percentage occupancy per year)	3.32 (0.23)	8340.5	**<0.001**	167.5	**<0.001**	1847.5	0.713	841.5	0.056
Advance in northern range margin (km northwards per year)	2.34 (0.31)	7314	**<0.001**	1218	0.169	1541	0.237	1054	0.569

Supplementary Table 3 | Details of supplementary analyses repeating the main interspecific analysis (Table 1), with alternative approaches. Two conservative approaches were tested; first, we used phylogenetic generalized least-squares (PGLS) models to test the relationship between change in emergence date and change in abundance, occupied distribution, and northern range margin (NRM), accounting for phylogenetic autocorrelation. Second, we tested these relationships using restricted subsets of data and generalized linear mixed-effects models (GLMMs), firstly excluding species which may, within the UK, be univoltine at some sites or in some years and multivoltine elsewhere (i.e. univoltine species which may have a second generation in warm years and/or at southern sites, or multivoltine species which may have only a single generation in cool years and/or at northern sites), and secondly analysing such flexible species in isolation. Finally, we tested the relationship between change in emergence date and change in abundance as before, using trends in emergence date and abundance calculated over the full time-period of available data (1973–2017) instead of limited to the study period (1995–2014). Test statistics (TS) and associated *P*-values are given for the effect of change in emergence date, or the two- or three-way interaction term in which it is contained; TS for PGLS models was F, and TS for GLMMs was the χ^2 value of a Likelihood Ratio Test. The results of the majority of re-analyses support the findings of the main analyses reported in Table 1, except as follows. Phylogenetically-constrained analysis of the relationship between phenological advances and changes in abundance was marginally non-significant when habitat specialisation class was included (significant in main analysis). Phylogenetically constrained analysis of the relationship between change in abundance and change in distribution size was non-significant when both voltinism and habitat specialisation class were included (significant in main analysis), but significant when only habitat specialisation class was included. The relationship between phenological advances and changes in northern range margin was marginally non-significant when only strictly univoltine or multivoltine species were included (significant in main analysis). Relationships between phenological advances and changes in abundance, distribution size and northern range margin were all non-significant for species which are flexibly univoltine or bivoltine within their UK range. Finally, the relationship between change in emergence date and change in abundance over longer time-scales was non-significant when habitat specialisation class was included (significant in main analysis).

				Overall model parameters			
Type of re-analysis	Dependent variable	Independent variable	Interacting covariates	n	AIC	R^2 (adjusted or marginal)	TS (*P*)
Phylogenetically-constrained	Change in abundance	Change in emergence date	Voltinism	130	-518.0	0.182	**4.04 (0.047)**
			Voltinism * Class	130	-526.8	0.258	2.58 (0.111)
	Change in occupied distribution		Voltinism	130	604.8	0.122	1.48 (0.227)
			Voltinism * Class	130	592.8	0.222	**8.14 (0.005)**
	Change in NRM		Voltinism	38	225.1	-0.043	0.98 (0.329)
			Voltinism * Class	38	218.4	0.197	**10.40 (0.003)**
	Change in occupied distribution	Change in abundance	-	130	553.5	0.399	**86.73 (<0.001)**
			Voltinism * Class	130	543.2	0.469	**5.97 (0.016)**
	Change in NRM		Voltinism	38	217.8	0.097	**4.99 (0.032)**

			Voltinism * Class	38	212.8	0.009	2.65 (0.114)
	Change in occupied distribution	Model- predicted change in abundance	Voltinism	130	254.4	0.003	1.39 (0.240)
			Voltinism * Class	130	254.4	0.053	**8.19 (0.005)**
	Change in NRM		Voltinism	38	111.0	0.109	**5.50 (0.025)**
			Voltinism * Class	38	111.0	0.081	**4.28 (0.046)**
Strict voltinism only	Change in abundance	Change in emergence date	Voltinism	94	-396.3	0.065	2.75 (0.097)
			Voltinism * Class	94	-399.2	0.176	**6.02 (0.014)**
	Change in occupied distribution		Voltinism	94	444.7	0.025	2.07 (0.151)
			Voltinism * Class	94	437.4	0.115	**9.23 (0.002)**
	Change in NRM		Voltinism	28	169.4	0.096	0.84 (0.361)
			Voltinism * Class	28	172.0	0.309	**5.26 (0.022)**
Flexibly univoltine-bivoltine species only	Change in abundance	Change in emergence date	Voltinism	36	-146.0	0.244	3.56 (0.059)
			Voltinism * Class	36	-144.8	0.284	N/A (rank-deficient data)
	Change in occupied distribution		Voltinism	36	133.8	0.081	0.09 (0.769)
			Voltinism * Class	36	141.4	0.077	N/A (rank-deficient data)
	Change in NRM		Voltinism	10	54.7	0.418	1.15 (0.283)
			Voltinism * Class	10	58.6	0.366	N/A (rank-deficient data)
Longer time-scale	Change in abundance	Change in emergence date	Voltinism	130	-644	0.062	**4.86 (0.028)**
			Voltinism * Class	130	-644	0.084	0.17 (0.680)

Supplementary Table 4 | Details of supplementary analysis repeating the main interspecific analysis (Table 1), with obligate univoltine species and functional univoltine species treated separately. We retested the relationship between change in emergence date and change in abundance, occupied distribution, and northern range margin (NRM), using generalized linear mixed-effects models. For this analysis, we treated voltinism as a three-level categorical variable (obligate univoltine, functional univoltine, and multivoltine), where species which have the capacity to be multivoltine somewhere within their geographic distribution but are functionally univoltine throughout Britain were assigned to a separate category from species which are univoltine throughout their global range. χ^2 values from a Likelihood Ratio Test and associated *P*-values are given for the effect of change in emergence date, or the two- or three-way interaction term in which it is contained. The results of these re-analyses support the findings of the main analyses reported in Table 1 in all cases.

			Overall model parameters				Effect sizes			
Dependent variable	Independent variable	Interacting covariates	n	AIC	R^2 (adjusted or marginal)	TS (*P*)	Subset of species	n	Effect size (s.e.)	X^2 (*P*)
Change in abundance	Change in emergence date	Voltinism	130	-549	0.139	**5.98 (0.050)**	Obligate univoltine	75	-0.01 (0.01)	0.24 (0.620)
							Functionally univoltine	16	-0.02 (0.03)	0.79 (0.370)
							Multivoltine	39	0.02 (0.01)	**10.60 (0.001)**
		Voltinism * Class	130	-551	0.225	**8.03 (0.018)**	Obligate univoltine habitat specialists	12	-0.09 (0.04)	**4.82 (0.028)**
							Obligate univoltine generalists	63	0.01 (0.01)	0.38 (0.540)
							Functionally univoltine habitat specialists	6	0.03 (0.10)	0.1 (0.760)
							Functionally univoltine generalists	10	-0.03 (0.03)	1.12 (0.290)
							Multivoltine habitat specialists	3	-	-
							Multivoltine generalists	36	0.02 (0.01)	**11.2 (<0.001)**
Change in occupied distribution		Voltinism	130	588	0.025	1.65 (0.440)	Obligate univoltine	75	-0.90 (1.10)	0.74 (0.390)
							Functionally univoltine	16	-0.12 (1.16)	0.008 (0.930)
							Multivoltine	39	0.59 (0.64)	0.89 (0.340)
		Voltinism * Class	130	584	0.072	4.60 (0.100)	Obligate univoltine habitat specialists	12	-5.93 (3.33)	3.45 (0.063)

							Obligate univoltine generalists	63	-0.05 (1.16)	0.008 (0.930)
							Functionally univoltine habitat specialists	6	1.31 (3.12)	0.18 (0.700)
							Functionally univoltine generalists	10	0.78 (1.51)	0.76 (0.380)
							Multivoltine habitat specialists	3	-	-
							Multivoltine generalists	36	0.49 (0.49)	1.07 (0.300)
Change in NRM		Voltinism	130	700	0.051	2.19 (0.330)	Obligate univoltine	75	-0.43 (1.83)	0.15 (0.700)
							Functionally univoltine	16	-1.31 (2.27)	0.38 (0.540)
							Multivoltine	39	1.77 (0.88)	**4.04 (0.044)**
		Voltinism * Class	130	702	0.138	**6.48 (0.039)**	Obligate univoltine habitat specialists	12	-3.34 (6.22)	0.53 (0.470)
							Obligate univoltine generalists	63	0.13 (1.82)	0.005 (0.940)
							Functionally univoltine habitat specialists	6	-5.61 (7.80)	0.52 (0.510)
							Functionally univoltine generalists	10	-0.89 (2.20)	0.20 (0.650)
							Multivoltine habitat specialists	3	-	-
							Multivoltine generalists	36	1.58 (0.74)	**4.56 (0.033)**

Supplementary Table 5 | Classification procedure for habitat specialisation. Moths were assigned as "habitat specialists" or "wider-countryside generalists" using these criteria, directly replicating the approach used to assign butterflies to the same categories in Asher *et al.*[1].

Habitat specialists	*Wider-countryside generalists*
Confined[1] to specific, discrete habitat 'islands' that are localized or patchy in the modern landscape[2] (i.e. species of downland, heathland, woodland clearings etc.)	Broad habitat requirements or use habitats that are widely distributed in the farmed countryside (e.g. generalist grassland, woodland or hedgerow species)
Rarely or never use linear habitats such as hedgerows or road verges	Can use linear habitats such as hedgerows and road verges
Usually only one or two species of larval foodplant	Often several species of larval foodplants
Relatively sedentary	Relatively mobile
Mostly have a single generation per year	Often multi-brooded

[1] Consider only specialization *within* a species' range if it is range-restricted. E.g. Scotch Argus *Erebia aethiops* is mainly restricted to northern Scotland, but its habitat requirements match those of a WC species within its range; therefore it would be assigned to WC. Additionally, if a species might be assigned differently in different parts of its range, consider which classification applies to the larger part of its range (e.g. *E. aethiops* might be considered a HS species in England only, but is a WC species when including Scotland).

[2] Consider the availability of specific habitat types. E.g. compare Purple Hairstreak *Favonius quercus* and Purple Emperor *Apatura iris*; both species associated with oak woodland. Colonies of *F. quercus* can persist on single, isolated oak trees, of which there are many in the wider countryside; therefore this is assigned to WC. By contrast, *A. iris* is mainly restricted to larger tracts of mature woodland with suitable sallow trees for larval hostplants, and is therefore assigned to HS.

Supplementary Table 6 | Relative frequencies of species assigned to various categories. Frequencies of species are given at each intersection of two categories (e.g. "univoltine butterflies"); frequencies given at the intersection of the same category twice are the total frequency of that category within the full dataset.

	**Butterflies**	**Moths**	**Univoltine species**	**Multivoltine species**	**Habitat specialists**	**Wider-countryside generalists**
**Butterflies**	**29**					
**Moths**	N/A	**101**				
**Univoltine species**	18	73	**91**			
**Multivoltine species**	11	28	N/A	**39**		
**Habitat specialists**	12	9	18	3	**21**	
**Wider-countryside generalists**	17	92	73	36	N/A	**109**

Supplementary References

1. Asher, J., Warren, M., Fox, R., Harding, P., Jeffcoate, G & Jeffcoate, S. *Millennium Atlas of Butterflies in Britain and Ireland*. (OUP Oxford, 2001).
