# Supplementary material for: Climate-induced phenology shifts linked to range expansions in species with multiple reproductive cycles per year
Source: Nat Commun. 2019 Oct 24;10:4455. doi: 10.1038/s41467-019-12479-w (PMC6813360; doi:10.1038/s41467-019-12479-w)
Supplement: Supplementary file 3 — Description of Additional Supplementary Files [file 41467_2019_12479_MOESM3_ESM.pdf]

Description of Additional Supplementary Files

File Name: Supplementary Data 1

Description: Species-level classifications and trends over the full study period for all 130 species included in the study. Categorical and descriptive variables are given as follows. DATA: phenology and abundance trends calculated from UK Butterfly Monitoring Scheme (BMS) or Rothamsted Insect Survey (RIS) data; TAXON: butterfly or moth; VOLTINISM: univoltine (1) or multivoltine (2) species within the UK range; STRICT_VOLTINISM: indicates whether VOLTINISM classification applies universally across global range (1) to the best of our knowledge, or may vary (0); CLASS: habitat specialist (HS) or wider-countryside generalist (WC), with classifications drawn from Asher et al. (2001) Millennium Atlas of Butterflies in Britain and Ireland for butterflies and the classification approach replicated for moths included in this study (Table S5); SCHEDULE41: species is listed under Schedule 41 of the Natural Environment and Rural Communities (NERC) Act 2006; NORTHERLY: species' northern range margin is within 100 km latitude of John O'Groats (TRUE), or northern range margin is reached further south than this latitude (FALSE); SITES: number of populations recorded by population monitoring schemes and included in this study; SITE.YEARS: total number of years of monitoring across all populations included in this study; RECORDS: total number of recording events (i.e. one transect or light-trap sample) in which at least one individual was recorded included in this study; INDIVIDUALS: total number of individuals recorded across all recording events included in this study; RECORDED.HECTADS: number of hectads in which the species was recorded in any year; HECTAD.RECORDS; total number of hectad-level presence records across all years. For each variable from emergence date (PHENO), abundance (ABUND), occupied distribution (DISTRIB) and northern range margin (MARGIN), the slope (SLOPE) of the trend over 20 years is given, alongside the standard error (SE) of that slope and the X2 (CHI) and P-values (P) of a significance test for the difference of that slope from zero. The slopes can be interpreted as follows. PHENO: annual advance in days; ABUND: annual change in abundance as a log(odds ratio); MARGIN: annual northwards advance in km; DISTRIB: annual percentage change in the proportion of recorded hectads that were occupied.

File Name: Supplementary Data 2

Description: Phylogeny used in phylogenetic generalized leastsquares (PGLS) models. The phylogeny was constructed for all 130 study species at the marker cytochrome c oxidase subunit I (COI), in Geneious version 11.1.4.
