# Supplementary material for: Climate-induced phenology shifts linked to range expansions in species with multiple reproductive cycles per year
Source: Nat Commun. 2019 Oct 24;10:4455. doi: 10.1038/s41467-019-12479-w (PMC6813360; doi:10.1038/s41467-019-12479-w)
Supplement: Supplementary file 6 — Reporting Summary [file 41467_2019_12479_MOESM6_ESM.pdf]

nature research

Corresponding author(s): Callum J. Macgregor
Last updated by author(s): Jul 25, 2019

Reporting Summary

Nature Research wishes to improve the reproducibility of the work that we publish. This form provides structure for consistency and transparency in reporting. For further information on Nature Research policies, seeAuthors & Referees and theEditorial Policy Checklist .

Statistics

For all statistical analyses, confirm that the following items are present in the figure legend, table legend, main text, or Methods section.

n/a	Confirmed	
☐	☒	The exact sample size (*n*) for each experimental group/condition, given as a discrete number and unit of measurement
☐	☒	A statement on whether measurements were taken from distinct samples or whether the same sample was measured repeatedly
☐	☒	The statistical test(s) used AND whether they are one- or two-sided<br>*Only common tests should be described solely by name; describe more complex techniques in the Methods section.*
☐	☒	A description of all covariates tested
☐	☒	A description of any assumptions or corrections, such as tests of normality and adjustment for multiple comparisons
☐	☒	A full description of the statistical parameters including central tendency (e.g. means) or other basic estimates (e.g. regression coefficient) AND variation (e.g. standard deviation) or associated estimates of uncertainty (e.g. confidence intervals)
☐	☒	For null hypothesis testing, the test statistic (e.g. *F*, *t*, *r*) with confidence intervals, effect sizes, degrees of freedom and *P* value noted<br>*Give P values as exact values whenever suitable.*
☒	☐	For Bayesian analysis, information on the choice of priors and Markov chain Monte Carlo settings
☒	☐	For hierarchical and complex designs, identification of the appropriate level for tests and full reporting of outcomes
☐	☒	Estimates of effect sizes (e.g. Cohen's *d*, Pearson's *r*), indicating how they were calculated

Our web collection on statistics for biologists contains articles on many of the points above.

Software and code

Policy information about availability of computer code

Data collection	No software was used in the data collection process (data were obtained from Centre for Ecology and Hydrology, Butterfly Conservation and Rothamsted Research as described in the Methods section). Identical datasets can be obtained from these sources on request.
Data analysis	All analyses were conducted in open-source software, R version 3.5.0, as stated in the manuscript. All R scripts, from initial processing of datasets to final analyses, are archived online at Zenodo (doi: 10.5281/zenodo.3351514).

For manuscripts utilizing custom algorithms or software that are central to the research but not yet described in published literature, software must be made available to editors/reviewers. We strongly encourage code deposition in a community repository (e.g. GitHub). See the Nature Research guidelines for submitting code & software for further information.

Data

Policy information about availability of data

All manuscripts must include a data availability statement. This statement should provide the following information, where applicable:
- Accession codes, unique identifiers, or web links for publicly available datasets
- A list of figures that have associated raw data
- A description of any restrictions on data availability

Datasets were obtained respectively from the UKBMS, Rothamsted Research (RIS) and Butterfly Conservation (BNM and NMRS), and may be requested from the same sources. All R scripts, from initial processing of datasets to final analyses, are archived online at Zenodo (doi: 10.5281/zenodo.3351514).

Field-specific reporting

Please select the one below that is the best fit for your research. If you are not sure, read the appropriate sections before making your selection.

Ecological, evolutionary & environmental sciences study design

All studies must disclose on these points even when the disclosure is negative.

Study description	We used data obtained by four recording schemes to assess changes in phenology, abundance, distribution size and latitude of the northern range margin for 130 species of British Lepidoptera over a 20-year period (1995-2014). We used generalised linear mixed-effects models to investigate the relationships between trends in each variable and two measures of life-cycle flexibility (voltinism and habitat specialization) at both between- and within-species levels. Phenological trend (i.e. advanced or delayed flight period) was treated as the independent variable, since this is directly influenced by climate change. Voltinism (i.e. number of generations per year) and habitat specialization were treated as covariates and were able to interact with phenological trend to determine the effect on the dependent variables, which were trends in abundance, distribution size and northern range margin.<br>Additionally, we used annual estimates of phenology and population size to investigate the population dynamics underpinning observed relationships between phenology and abundance trends, using a similar modelling approach.
Research sample	The research sample consists of long-term data (over the period 1995-2014) from four existing datasets (the UK Butterfly Monitoring Scheme, the Rothamsted Insect Survey, Butterflies for the New Millennium, and the National Moth Recording Scheme) on 130 species (29 butterflies and 101 moths). Collectively, these datasets provide information on the abundance, phenology and distribution of butterflies and moths in Great Britain. The 20-year time period was selected to maximise the information content of the dataset; we could not obtain more recent data from all four of the datasets, and the BNM contains fewer records from years prior to its commencement in 1995. From the set of all available data within this time period, we excluded species according to a range of criteria (described in full in "Data selection", line 256-328) relating both to their ecology (i.e. species which might have atypical responses to climate change were excluded) and the data on them (i.e. species with too few observations or unreliable data were excluded). This left 130 species, and we used data from all 130 species spanning the full time period for the study.
Sampling strategy	No sample-size calculation was performed. Across the four datasets, we had access to a vast quantity of data and focussed our sampling strategy on ensuring we made use of the highest-quality data. From these datasets, we selected 422913 abundance records (representing 3472675 individual Lepidoptera), and 913037 hectad-level distribution records of species presence.<br>When selecting which species to include in the study according to data availability, we excluded populations of species if they had been recorded in fewer than 15 years between 1995-2014 (from a maximum of 20 years possible recording), and then excluded species if they had been recorded at fewer than three populations. This meant that all species-level trends over time in phenology and abundance were calculated from at least 45 observations, with at least 3 levels of a random effect (random intercepts) and at least 15 observations per level. We deemed this sufficient to estimate a trend over time in each variable. The resultant sample size of 130 species was an emergent property of the underlying data and these sampling criteria. We deemed n = 130 to be a sufficient sample for generalised linear mixed-effects models with a relatively simple structure and only three independent variables.
Data collection	Data were collected for each recording scheme by volunteers, following the respective procedures of each scheme.
Timing and spatial scale	We used data collected between 1995 and 2014, within Great Britain (i.e., UK excluding Northern Ireland and the Channel Islands).
Data exclusions	Once the final dataset of 130 species had been established (with species excluded following pre-established criteria, as described above and in the methods section lines 256-328), no further data were excluded from analyses.
Reproducibility	Given that the data used can be obtained by application to the same recording schemes, and that we have archived all R scripts, it should be possible for our results to be reproduced exactly.
Randomization	In our multi-species analyses, data were grouped according to taxon (i.e. butterfly or moth) because of potential differences in recording methods, rather than biological differences in these taxa. Taxon group was included in models as a random effect (random intercepts). In analyses of annual data (as opposed to trends over time), data were grouped according to population (i.e. recording site) to account for spatial autocorrelation between observations made of the same population in different years. Population was included in models as a random effect (random intercepts).
Blinding	Blinding was not relevant during data collection because we used secondary data from recording schemes that were not explicitly collected for the purposes of phenological analysis. Data selection was conducted procedurally (as described above) and therefore species inclusion was based solely on data quality, and not biased by other factors.

Did the study involve field work? ☐ Yes ☒ No

Reporting for specific materials, systems and methods

We require information from authors about some types of materials, experimental systems and methods used in many studies. Here, indicate whether each material, system or method listed is relevant to your study. If you are not sure if a list item applies to your research, read the appropriate section before selecting a response.

Materials & experimental systems

n/a	Involved in the study
☒	☐ Antibodies
☒	☐ Eukaryotic cell lines
☒	☐ Palaeontology
☒	☐ Animals and other organisms
☒	☐ Human research participants
☒	☐ Clinical data

Methods

n/a	Involved in the study
☒	☐ ChIP-seq
☒	☐ Flow cytometry
☒	☐ MRI-based neuroimaging
